# The Cleveland Clinic Way

**DOI:** 10.1093/jhps/hnv068

**Published:** 2015-11-20

**Authors:** Austin V. Stone, Allston J. Stubbs

**Affiliations:** Department of Orthopaedic Surgery, Wake Forest School of Medicine, Winston-Salem, NC 27157-1070, USA ausstone@wakehealth.edu; astubbs@wakehealth.edu

The global healthcare market continues to rapidly evolve, so it is important for all healthcare practitioners to be equally adept in their management of healthcare delivery as in their practice of medicine. A recent book by Toby Cosgrove MD, the CEO and president of the Cleveland Clinic, offers exquisite insight into transforming the institution’s healthcare delivery and outcomes to meet the 21st century expectations of patients and payors. The book, *The Cleveland Clinic Way: Lessons in Excellence from One of the World’s Leading Healthcare Organizations* (MgGraw-Hill, 2014), describes the journey of modernizing a large academic institution. This project encompassed all practice areas from personnel organization to global expansion, from outcomes reporting to centers of excellence, and from cost-effective medicine to patient satisfaction. Dr Cosgrove offers a unique perspective on the business of medicine and offers valuable pearls of wisdom in the execution of value-based medicine.

The contents of the book are logically developed to explore the opportunities for care efficiency and efficacy in large group practices. The principles of optimizing care delivery through economies of scale may be helpful to smaller practices negotiating with hospitals, surgical centers and suppliers. Electronic medical records (EMRs) are becoming universal and offer additional opportunities to improve care collaboration. As Dr Cosgrove discusses collaborative care may be more efficient in managing patients, especially those that require subspecialty. This principle is immediately applicable to those who provide such specialized services as hip preservation surgery, since patients will often receive work-up and long-term follow-up by primary care physician-based teams.

Cost-control pressures are ubiquitous but inexpensive care does not necessarily yield optimal societal or patient outcomes. Dr Cosgrove argues that EMRs’ utility may be leveraged to not only improve the efficiency of patient care management but also offer substantial value for improving outcomes. EMRs can predict potential medical hazards to patients, allow for rapid querying of patient data and the ability to enhance transparency in patient, surgeon and practice outcomes. His arguments are supported by detailed case studies that demonstrate using EMRs for transparency in outcomes; outcomes reporting can be a surgeon’s best asset rather than a medical and financial liability. Under his leadership at the Cleveland Clinic, this strategy has reduced costs and increase revenue while increasing patient satisfaction and clinical outcomes.

*The Cleveland Clinic Way* ultimately makes a case for patient-centric care that is derived as much from careful attention to operations and business practices as it is from medical knowledge and skill. The innovations in healthcare delivery outlined in his book are supported by detailed case studies from a wide breadth of literature. His perspectives are easily digested and universally applicable to any practicing physician or surgeon.

## CONFLICT OF INTEREST STATEMENT

None declared.

